# Geometric Analysis of Type B Aortic Dissections Shows Aortic Remodeling After Intervention Using Multilayer Stents

**DOI:** 10.3390/ma13102274

**Published:** 2020-05-15

**Authors:** Victor S. Costache, Jorn P. Meekel, Andreea Costache, Tatiana Melnic, Crina Solomon, Anca M. Chitic, Cristian Bucurenciu, Horatiu Moldovan, Iulian Antoniac, Gabriela Candea, Kak K. Yeung

**Affiliations:** 1 NextCardio Project, Lucian Blaga University of Sibiu, 550024 Sibiu, Romania; victorscostache@gmail.com (V.S.C.); jornmeekel@gmail.com (J.P.M.); melnictatiana.85@gmail.com (T.M.); crinasol@yahoo.com (C.S.); ancachitic@yahoo.com (A.M.C.); cristianbucurenciu@gmail.com (C.B.); candeagabriela@gmail.com (G.C.); kakkhee.yeung@gmail.com (K.K.Y.); 2 Department of Vascular Surgery, Amsterdam University Medical Centers, 1105 AZ Amsterdam, The Netherlands; 3 Surgery Department, Zaans Medisch Centrum, 1502 DV Zaandam, The Netherlands; 4 Department of Cardiac Surgery, Titu Maiorescu University, 040441 Bucharest, Romania; h_moldovan@hotmail.com; 5 Materials Science and Engineering Faculty, University Politehnica of Bucharest, 060042 Bucharest, Romania; antoniac.iulian@gmail.com

**Keywords:** multilayer flow modulator, aortic dissection, aortic remodeling, computational fluid dynamics, geometric analysis

## Abstract

Recently, multilayer stents for type B aortic dissections (TBAD) have been proposed to decrease false lumen flow, increase and streamline true lumen flow, and retain branch vessel patency. We aimed to provide a protocol with standardized techniques to investigate aortic remodeling of TBAD by multilayer flow modulators (MFM) in static geometric and hemodynamic analyses. Combining existing literature and new insights, a standardized protocol was designed. Using pre- and postoperative CT scans, geometric models were constructed, lumen dimensions were calculated, computational fluid dynamics (CFD) models were composed, and velocity and pressures were calculated. Sixteen TBAD cases treated with MFM were included for analysis. For each case, aortic remodeling was analyzed using post-processing medical imaging software. After 3D models were created, geometrical anatomical measurements were performed, and meshes for finite element analysis were generated. MFM cases were compared pre- and postoperatively; true lumen volumes increased (*p* < 0.001), false lumen volumes decreased (*p* = 0.001), true lumen diameter at the plane of maximum compression (PMC) increased (*p* < 0.001), and false lumen index decreased (*p* = 0.008). True lumen flow was streamlined, and the overall fluid velocity and pressures decreased (*p* < 0.001 and *p* = 0.006, respectively). This protocol provided a standardized method to evaluate the effects of MFM treatments in TBAD on geometric analyses, PMC, and CFD outcomes.

## 1. Introduction

Medical treatment is still the preferred treatment option in many centers for the treatment of acute type aortic B dissection (TBAD). However, pharmacological treatment fails to induce aortic remodeling with progression to aneurysm formation requiring aortic intervention within seven years in nearly 40% [1]. Now according to the DISSECT criteria, thoracic endovascular aortic repair (TEVAR) has been advocated as standard care for acute TBAD [2].

However, stent graft-induced new entry tears into the distal thoracic or abdominal aorta can arise as a complication of the TEVAR during the treatment of acute TBAD [3,4]. Besides, aortic remodeling after TEVAR for TBAD is still questionable as aortic growth or new aneurysm occurs in 7% to 84% of the cases for the thoracic aorta and 10% to 54% of the cases for the abdominal aorta [5,6,7,8,9]. Newer techniques, including fenestrated or branched TEVAR, and hybrid approaches were proposed as alternatives to open surgery or conventional TEVAR in complex cases involving branch arteries. The latter therapies demonstrate prolonged learning curves, higher costs, and delays due to manufacturing, and the results of long-term follow-up are not available yet [10,11].

Another alternative to traditional TEVAR is the multilayer flow modulator stent (MFM^®^ Cardiatis, Isnes, Belgium), which is a self-expanding cobalt alloy bare-metal stent with interrelated wires in three to five layers. MFMs have been designed to be used in complex endovascular aortic repairs, mainly for thoracoabdominal aneurysms. Specific characteristics of the MFM stents include (i) laminar blood flow with less wall shear stress; (ii) no necessity for extra procedures to vascularize branch arteries; (iii) possibility to overlap to form complex structures; (iv) high resistance to kinking and fatigue; (v) the possibility of custom MFM construction to match patient’s anatomy [12,13]. In clinical research settings, the MFM has been used to treat chronic symptomatic aortic dissection [11].

Endovascular aortic repair with multilayer stents is a promising treatment for complicated TBAD due to the unique ability of these devices to stabilize the entire aortic wall without compromising the flow in the major aortic side branches.

Geometric morphological analyses have previously been used to evaluate the remodeling of the aorta after endovascular interventions for TBAD [11,14,15]. These measurements mainly involve true lumen (TL) and false lumen (FL) dimensions, including the well-recognized false lumen index (FLI). Finotello et al. emphasized the importance of the use of new geometric measurements in the follow-up of patients treated with MFM stent in abdominal aortic aneurysm (AAA) [16]. Recently, following the use of MFM stents for TBAD, measurements of the plane of maximum compression (PMC) have been introduced [17], in which the maximum amount of compression on the true lumen is applied as a consequence of the aortic dissection as an additional parameter besides the FLI. To study the hemodynamic effect more extensively, computational fluid dynamics (CFD) might provide a proper complement to geometric analyses [18,19,20,21,22]. 

The aim of this study was to study the flow and remodeling of the true and false lumens in TBAD before and after endovascular treatment with MFM stents in our patient population. Geometric, PMC, and CFD results of MFM use for TBAD were presented.

## 2. Materials and Methods

### 2.1. Ethics Statement

This study was approved by the Department for Clinical Trials and Medical Infrastructure of the Romanian Ministry of Health (study EU Protocol v4.0 number DM4948/37). The research was carried out in accordance with the Code of Ethics of the World Medical Association (Helsinki Declaration) of 1975, as revised in 1983. The implementation of the presented study was supervised by the Ethical Committee of the Lucian Blaga University of Sibiu and by the Ethical Committee of Polisano European Hospital (Sibiu, Romania). Starting from May 2018, the new rules defined in the EU General Data Protection Regulation (GDPR) were embraced and respected in the study.

### 2.2. Study Design, Patient Selection, and Follow-Up

From a prospective single-center MFM registry cohort, the anonymized CT scans of 16 patients with TBAD who underwent endovascular repair using MFM stent between April 2014 and February 2018 were consecutively included in the Polisano European Hospital, Sibiu, Romania. CT scans were collected preoperatively, directly postoperatively, and at the latest follow-up moment (varying from 1 to 36 months postoperatively). A study protocol was formulated by the authors to perform geometric analyses of TL and FL, PMC, FLI, and CFD.

Inclusion criteria in the study were: (i) primary type B aortic dissections and residual type B dissections following type A dissection repair; (ii) type B aortic dissections identified by angio-CT scan (acute, subacute, and chronic); (iii) patient age between 18 and 85 years; (iv) non-pregnant patients; (v) patient was available for follow-up.

Although the use of the MFM for aortic dissections is outside instruction-for-use (IFU), the IFU, as established for aortic aneurysms, was utilized. These included: (i) a minimum two-centimeter landing zone in non-diseased aortic wall proximally and distally to the intended placement zone of the MFM; (ii) aneurysm diameter below 65 millimeters, if present; (iii) MFM oversizing of 15–25% in the landing zones compared to the aortic wall; (iv) in overlapping stents, the proximal stent should have a smaller diameter and should be deployed first. Hereafter, the larger distal stent was deployed. The overlap of the stents should be at least six centimeters for straight aortas and eight centimeters for angulated aortas.

### 2.3. CTA Imaging Protocol

CTA scanning was performed in the supine position using a 128-row multi-slice scanner (Somatom Definition AS, Siemens, Munich, Germany). CT scans covered the full extension of the dissection. DICOM files were obtained with a slice thickness of 0.6 mm and a pixel size of 0.625 × 0.625 mm. 

### 2.4. Measurement Protocol

#### Segmentation and Geometrical Reconstruction of the Aorta

Surface reconstruction of the aortic dissection was accomplished using MIMICS (v20.0; Materialise, Leuven, Belgium) postprocessing software. Within MIMICS, a standardized protocol for image segmentation was performed for each CT scan to create 3D aortic models representing the false and true lumen. The contrast-enhanced CT scans in digital imaging and communications in medicine (DICOM) format were imported into MIMICS. Firstly, the Hounsfield Units threshold was set at a minimum of 175. This threshold functionality offers clear visualization of contrast-enhanced anatomy (thoracic aorta and bone) by allowing the low-attenuation anatomy (such as soft tissue) to be removed.

**Figure 1:**
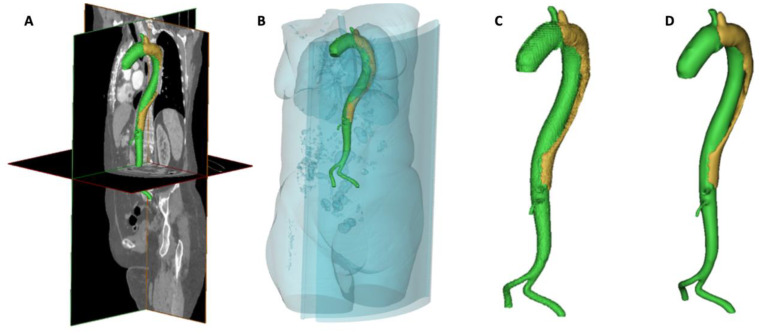
Within MIMICS, axial slices were converted into a 3D model using a Hounsfield Units cutoff value of 175. Hereafter, the ascending aorta, aortic arch, descending aorta, abdominal aorta, bifurcation, and proximal iliac arteries were isolated (**A**–**C**). True and false lumens were smoothened and separated for further analyses (**D**).

Consequently, automated software functions, including “Region growing”, “Calculate 3D”, “Crop Mask”, “Edit Mask”, and “Dynamic Region Growing”, were executed. Hereafter, the aorta was isolated (Figure 1A–C), and the model was smoothened and meshed, with an isolated true and false lumen (Figure 1D). Following 3D surface rendering, automatic and manual segmentation methods were used to visualize only the aorta. The aortic model was exported as a stereolithography (STL) format, providing a 3D triangular mesh (the surface contours of the model were approximated with a connected series of triangular faces).

True and false lumens were distinguished in MIMICS based on colors (green for true lumen and red for false lumen) to demonstrate the dimensions of both lumens. Hereafter, the geometrical evaluations of true and false lumen volumes, true lumen diameters at PMC, and FLI were performed pre- and post-procedurally and during the latest follow-up. FLI was calculated using the following formula, by dividing the false lumen volume to the volume of the true lumen.

false lumen volume index = false lumen volume/true lumen volume(1)

### 2.5. 3D Model Construction

The geometrical reconstruction of the aorta was transferred to 3-Matic (v13.0; Materialise, Leuven, Belgium) post-processing software to create a clean 3D model. Entry and re-entry tears between true and false lumens were created in 3-Matic conform contrast continuation, and size as observed in the CTA. Subsequently, an STL-file was created.

### 2.6. Computational Fluid Dynamics Modeling Protocol

CFD modeling was used to study hemodynamics in aortic dissections since flow, turbulence, and blood-wall-endoprosthesis interactions play a key role in the pathology and treatment of aortic dissections. Therefore, the STL-file was imported in Ansys Workbench (v18.2, ANSYS Inc., Canonsburg, PA, USA). After using the “Generate Part Names” function, aortic geometry was shown as “Solid Full Display”. Next, the surface was altered using the “Segment/Trim Surface” option and “By Angle Method” with an angle of 35°. The most proximal part of the aorta was identified as the “Inlet” in the “Create Part DEZ”, while all the branches were identified as “Outlets” in the “Create Part DEZ”. Afterward, curves were created using the “Extract Curves from Surfaces” option. The aortic wall itself was deselected, while the inlet and outlet surfaces were selected using the “Select All Appropriate Visible Objects” option. The “BSpline” function was chosen, and new curves were created. Now, for the aortic wall, the option “WireFrame Simple Display” was used. 

Next, using the “Create Body” option, fluid (i.e., blood) was added to the model. The “Centroid of 2 Points” function was selected, and, using the mouse, two 3D locations within the aortic wall were selected to instruct ANSYS to create the fluid part. Subsequently, the aortic wall was again fully displayed, using the “Solid Full Display” option. Now the “Global Mesh Size” option was used, including the following configuration: “Scale Factor” = 0.5, “Max Element” = 2, “Curvature/Proximity Based Refinement” should be enabled with “Min. Size Limit” = 0.5, “Elements in Gap” = 1, and “Refinement” = 18. The remainder of the parameters were retained in default settings.

Now, the mesh was created using the “Volume Mesh” option. The next settings were used: “Mesh Type” = “Tetra/Mixed”, “Mesh Method” = “Robust (Octree)”, and “Create Prism Layers” was disabled. Following, the “Compute” button was chosen. The mesh was now examined using “Surfaces” and “Solid and Wire” functions. The prism layer mesh contained orthogonal prismatic cells, normally reside next to wall boundaries. The geometry and mesh were exported in “Select Solver” under “Output” using the following settings: “Output Solver” = “ANSYS Fluent”. In “Boundary Conditions” under “Output”, the right boundaries were selected: aortic wall = “Wall”, proximal aortic inlet = “Velocity-inlet”, fluid or blood = “Fluid”, and distal branches = “Pressure-outlet”. Finally, the aortic flow model was exported, and the link between ICEM CFD and Fluent Set-up was created.

In “Set-up”, “Scale” was set at 0.001 (to convert meters into millimeters) for “X, Y, and Z Scaling Factor”, and default settings were retained for the remainder of parameters. The specifications of the fluid were converted from default air into the blood, by changing “Density” to 1060 kg/m^3^ and overwriting the specifications of the fluid. The “Inlet Velocity” was set at 0.35 m/s (average velocity of the fluid for healthy patients), and the “Outlet Pressure” at 0 Pa (a steady-state was used). Altogether, the following “Reference Values” were entered: “Area”: 1 m^2^, “Density”: 1060 kg/m^3^, “Enthalpy”: 0 j/kg, “Length”: 300 mm, “Pressure”: 0 pascal, “Temperature”: 309.15 K (=36 °C), “Velocity”: 0.35 m/s, “Viscosity”: 0.0035 kg/m-s, and “Ratio of Specific Heats”: 1.4. The calculations were running with 10 static iterations, and, for the Solution, another 10 static iterations were run. The results of the Solution were linked from the Fluent menu. To represent overall fluid velocity and overall fluid pressure, Streamline and Contour functions in ANSYS were used, respectively.

### 2.7. Workstation

All segmentation steps, geometrical reconstruction, and CFD analyses were performed using a high-speed workstation (64-bit Lenovo Desktop, CPU: Intel Xeon CPU E3-1280 v5 @ 3.70 GHz (8 cores); RAM Memory: 32GB; and Video Card: NVIDA Quadro M4000 (8GB GDDR5 + 16GB Shared Memory).

### 2.8. Statistical Analysis

For the presentation of the cases: continuous variables were expressed as mean ± standard deviation when normally distributed and as median and interquartile range when skewed. Categorical variables were expressed as percentages. Wilcoxon signed-rank test was used to compare preoperative and postoperative outcomes. Of all variables, a percental difference between pre- and directly postoperative was calculated. Hereafter, the two-tailed Spearman Rank Test was performed to assess the relationship between geometric and CFD results. All analyses were performed with SPSS (version 25.0; IBM Corporation, Somers, NY, USA). The *p*-values <0.05 were considered statistically significant. 

## 3. Results

### MFM Cases Analysis

**Figure 2:**
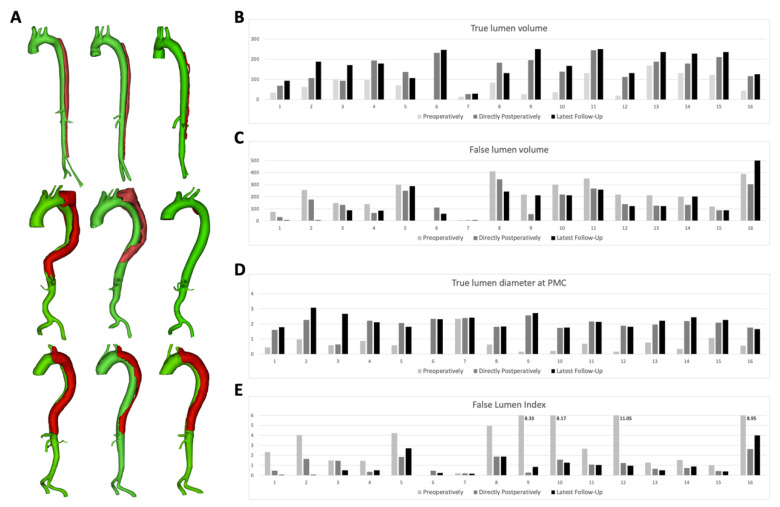
(**A**) Morphologic analysis of the primary three symptomatic chronic type B aortic dissection cases with the longest follow-up obtained from CT scans, preoperative, postoperative, and at the latest follow-up. All geometries comprised aortic branches and true and false lumens. Pre-op indicates preoperative; post-op, postoperative; FU, follow-up. The alterations in true lumen volume (**B**), false lumen volume (**C**), true lumen diameter at the plane of maximum compression (**D**)**,** and the false lumen index (**E**) of all 16 symptomatic chronic type B aortic dissections obtained from CT scans, preoperative, postoperative, and at the latest follow-up. PMC indicates the plane of maximum compression.

In this study, CT scans of 16 cases were included in for which the MFM stent was used as an intervention for TBAD. These 16 patients with complicated TBAD underwent endovascular repair using new generation MFM stents: two patients acute TBAD, six patients sub-acute TBAD, and eight patients chronic TBAD. All MFMs were placed in the true lumen. Detailed 3D reconstructions of the primary three cases with the longest follow-up are presented in Figure 2A, to illustrate preoperative, postoperative, and latest follow-up (median follow-up 12 months) aortic morphology. The number of months for the follow-up for each patient can be found in Table 1; the shorter follow up was 1 month (for patient 15), and the longest follow-up was 36 (for patients 1, 2, 3, and 9).

Table 2 presents pre- and postoperative volume measurements for true and false lumen, true lumen diameters at PMC, and FLI. True lumen volumes were compared pre- and postoperatively per case (Figure 2B). Analysis using MIMICS software showed a median increase of true lumen volume from 71.09 cm^3^ (IQR 34.95–110.89 cm^3^) preoperatively to 83.26 cm^3^ (IQR 111.31–194.57 cm^3^) directly postoperatively (*p* < 0.001). Overall, a mean increase of 157% was observed. During the latest follow-up visit, the median true lumen volume increased to 174.17 cm^3^ (IQR 129.31–234.98 cm^3^, *p* < 0.001). Compared to the preoperative situation, a mean increase of 201% was observed. In all studied cases, true lumen volume increased at the latest follow-up, with a maximum increase at case no. 9 (954%) and a minimum increase at case no. 13 (140%). 

Figure 2C shows the difference in false lumen volume comparing the pre-, postoperative, and latest follow-up morphological situation. A median decrease of false lumen volume from 218.30 cm^3^ (IQR 143.76–299.49 cm^3^) preoperatively to 133.69 cm^3^ (IQR 83.43–224.91 cm^3^) directly postoperatively (*p* < 0.001) was observed, with an overall mean decrease of 26%. During the latest follow-up visit per case (median follow-up 12 months), the median false lumen volume decreased to 123.42 cm^3^ (IQR 80.17–219.95 cm^3^, *p* = 0.001). 

When comparing the total volume of the aorta for the preoperative and postoperative, a mean decrease of 7.21% was observed; while comparing the preoperative and latest follow-up, a mean decrease of 17.36% was observed (see Table 1).

True lumen diameter at the PMC was compared pre- and postoperatively per case (Figure 2D). A median increase of true lumen diameter at the PMC was observed from 0.60 cm (IQR 0.40–0.82 cm) preoperatively to 2.07 cm (IQR 1.79–2.21 cm) directly postoperatively (*p* < 0.001). Overall, a mean true lumen increase of 374% was observed. During the latest follow-up visit, the median true lumen diameter at the PMC increased to 2.18 cm (IQR 1.80–2.41 cm, *p* < 0.001). Compared to the preoperative situation, a mean increase of 412% was observed.

Examination of the FLI ratio demonstrated a median decrease of 2.68 (IQR 1.46–6.56) preoperatively to a median value of 0.92 (IQR 0.45–1.59, *p* < 0.001) directly postoperatively. Within the 16 studied cases, FLI decreased for all cases, with a mean value of 60%. Figure 2E shows the difference in FLI comparing the pre-, postoperative, and latest follow-up morphological situation. During the latest follow-up visit per case (median follow-up 12 months), the median FLI decreased to 0.69 (IQR 0.35–1.10, *p* = 0.008). Compared to the preoperative situation, a mean decrease of 67% was observed. Case no. 1 presented the maximum decrease of FLI ratio during the latest follow-up (100%), and case no. 7 the minimum decrease (27%).

**Figure 3:**
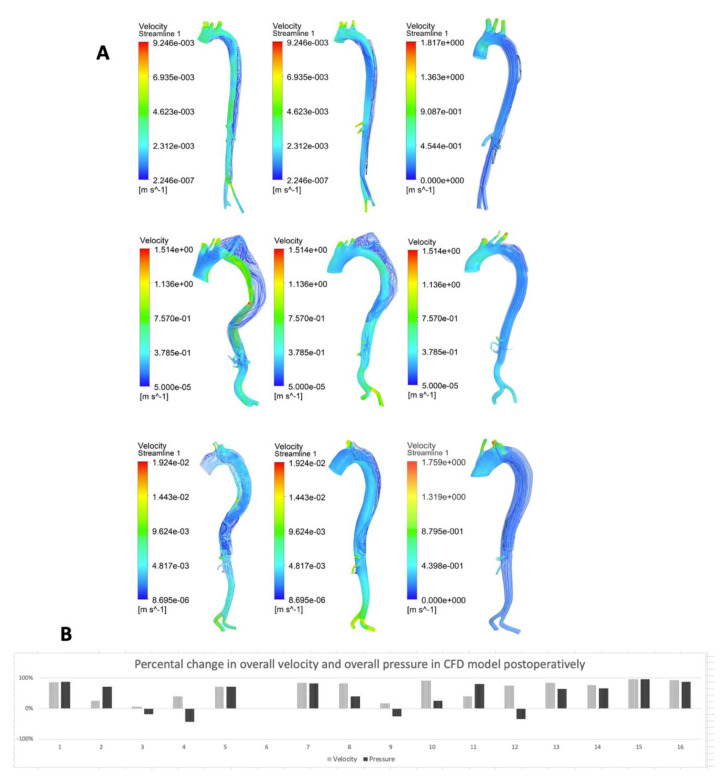
(**A**) Velocity maps of computational fluid dynamics (CFD) analyses of the primary three symptomatic chronic type B aortic dissection cases with the longest follow-up obtained from CT scans, preoperative, postoperative, and at the latest follow-up. All geometries comprised aortic branches and true and false lumens. Pre-op indicates preoperative; post-op, postoperative; FU, follow-up. (**B**) This figure presents the alterations in overall velocity and overall pressure calculated in the CFD models postoperatively per case.

Figure 3A shows the outcomes of the qualitative CFD analyses in ANSYS. Qualitative alterations in both false and true lumen flow were shown directly after surgery and during follow-up. In all cases, an increase of straightened intraluminal flow was observed (darker blue and increasingly aligned), with a concomitant decrease of turbulent flow in the false lumen. Overall fluid velocity and fluid pressures were calculated using ANSYS post-processing functions. A median percentage decrease of overall fluid velocity was observed of 77.8% (IQR 40.0–85.0%, *p* < 0.001) postoperatively. A median percentage decrease of overall fluid pressures was observed of 66.7% (IQR 3.1–81.1%, *p* = 0.006) postoperatively (Figure 3B).

The correlation analysis of preoperative and directly postoperative geometric and CFD results showed a strong positive correlation between true lumen volume change and true lumen diameter at PMC change (CC 0.618, *p* = 0.014) and a strong negative correlation between true lumen volume change and FLI change (CC −0.754, *p* = 0.001). Furthermore, moderate negative correlations were found between false lumen volume change and true lumen diameter at PMC change (CC −0.536, *p* = 0.040) and false lumen volume change and CFD pressure change (CC −0.582, *p* = 0.023). A negative correlation was also found between true lumen diameter at PMC change and FLI change (CC −0.746, *p* = 0.001), and a moderate positive correlation was found between true lumen diameter at PMC change and CFD pressure change (CC 0.571, *p* = 0.026).

## 4. Discussion

Our study provided a standardized protocol for geometrical and hemodynamic analysis for TBAD and aortic remodeling in the follow-up period. 

Recently, we and others presented early experiences using the new generation multilayer stent for endovascular treatment of aortic dissection as a possible solution for treating TBAD. Contrarily to traditional TEVAR for TBAD using stent-grafts, this endovascular approach with multilayer stents has a low risk of paraplegia, renal failure, stent-graft-induced new entry tears, and distal progression of the dissection with aneurysm formation through stabilization of the entire aorta with no covered fabric [11,23,24,25]. 

This prospective study included 16 patients with complicated type B aortic dissection that underwent endovascular repair using new generation MFM stents: two patients acute TBAD, six patients sub-acute TBAD, and eight patients chronic TBAD. The results suggested that the new generation MFM could expand the true lumen with a low risk of complications.

Our presented protocol analyzed PMC to assess decompression of the true lumen and qualitative and quantitative CFD analyses to study flow and remodeling of the true and false lumens in TBAD before and after treatment using MFM. 

The plane of maximum compression (PMC) is clinically relevant in the light of hemodynamic alterations and wall stress [26]. Results of this study showed that, at the latest available follow-up, there was a mean increase of 412% in true lumen diameter recorded at the plane of maximum compression. Only in one patient, no increase was observed. The well-recognized false lumen index showed a decrease in all cases (100%), which also progressively continued during follow-up, proving positive aortic remodeling and the efficacy of MFM in these cases. 

The concomitant increase of intraluminal flow and decrease of turbulent flow (and hereby flow vortices on the wall) in false lumen was suggested to induce positive shears stress, which could lead to endothelialization in the MFM and co-occurring of thrombosis of the false lumen. Interestingly, this theory is supported by the fact that these processes progress in the postoperative period [11,27,28]. The overall decrease of fluid velocity could also be explained by the straightened increased intraluminal flow since the overall velocity was calculated by the sum of all of the flow in all different directions in both true and false lumens. Furthermore, the fluid pressures decreased since the flow was straightened and mainly routed through the true lumen, leading to a decreased resistance.

Although the group was small, the positive and negative outcomes in the correlation analyses illustrated: (i) the beneficial effect of measuring the alterations in the diameter at the PMC as it correlated with both false and true lumens; (ii) the simultaneous enlargement of the true lumen and decrease of the false lumen as an effect of the endovascular procedure; (iii) the relationship that could be found between the geometric and CFD analyses.

The assessment of true and false lumen diameters and measured volumes showed an increase, respectively decreased, in the percent compared for each patient to the preoperative outcome, postoperative outcome, and the latest follow-up. The length of the longest follow-up varied for each patient from 1 month to 36 months (see Table 1).

Analysis data from Table 2 and Table 1 showed that certain patients fared worse than others, such as patients 5, 9, 14, and 16. The reason for these patients, except patient 16, was a genetic condition (Marfan disease), diabetes mellitus type 2. Looking for other elements that could influence the evolution after the intervention, we did not find others. However, we could outline that three patients out of 16 were smokers, and 10 patients out of 16 were with hypertension. We also thought that factors, such as length of aortic coverage and timing of treatment, might explain the variation seen in those patients.

The main aim was to produce a standardized protocol to analyze aortic remodeling in TBAD. However, we were aware of the limitations by presenting our geometrical analysis and hemodynamic changes after MFM stents placement in TBAD in this small case series. A discussion can be made that it could be only the mechanical effect of the expansion of the true lumen by a “bare” stent of any type, as we did not have a control group or other analyses of treatment with covered stents or no stents. Therefore, we encourage others to use this standardized protocol to compare data, as the protocol would be applicable for evaluations of other endovascular devices as well. 

The segmentation software was used by bio-engineers to analyze the geometrical cardiovascular anatomy and compute parameters. A limitation, specifically considering the protocol, is the lack of scientific input from different experts from different fields and backgrounds, as could be organized as a Delphi method discussion. Nonetheless, the current protocol could already be used to evaluate the efficacy of endovascular devices and might be improved in future versions. Potential changes in future work might include: (i) changes in the geometry of the aorta could be investigated using the prism layers for the mesh exports inside the ANSYS software; (ii) a transient state will be studied instead of steady-state for outlet pressure. The relatively short and variable follow-up length demands further studies with larger patient groups, followed over a longer period. Additionally, in future studies, alternative analysis methods, such as finite element analysis and computational fluid dynamics, should be undertaken. 

The authors conclude that the current protocol can be used to evaluate the effects of the multilayer stent and potentially other endovascular devices on geometric analyses, PMC, and both qualitative and quantitative CFD outcomes. Further research is needed to evaluate the efficacy and clinical effects of multilayer stents used for the endovascular treatment of thoracoabdominal aortic dissections.

## Figures and Tables

**Table 1:** The length of follow-up time, total volumes of aorta preoperative, postoperatively, and at the latest follow-up.

Cases	Pre-Op Aorta Vol. (cm^3^)	Post-Op Aorta Vol. (cm^3^)	Latest Follow-Up Aorta Vol. (cm^3^)	No of Months-Follow-Up
1	110.34	99.69	94.00	36
2	319.80	283.88	189.61	36
3	248.70	225.90	258.35	36
4	235.32	261.38	264.38	24
5	370.89	385.22	393.14	30
6	n/a	341.36	308.14	12
7	16.33	32.55	33.15	24
8	495.22	526.99	375.65	12
9	244.51	252.79	461.74	36
10	335.78	355.00	377.14	12
11	482.68	511.44	510.38	6
12	238.14	251.79	255.32	6
13	380.53	314.23	357.82	6
14	334.23	312.83	428.51	6
15	242.27	299.27	324.20	1
16	431.65	418.88	624.43	12

**Table 2:** Measurements preoperative, postoperatively, and at the latest follow-up.

Cases	Pre-Op				Post-Op				Latest Follow-Up			
	TL vol. (cm^3^)	FL vol. (cm^3^)	TL at PMC ^1^ (cm)	FLI	TL vol. (cm^3^)	FL vol. (cm^3^)	TL at PMC ^1^ (cm)	FLI	TL vol. (cm^3^)	FL vol. (cm^3^)	TL at PMC ^1^ (cm)	FLI
1	33.29	77.05	0.44	2.31	68.29	31.4	1.61	0.46	93.32	0.68	1.79	0.01
2	63.4	256.4	0.96	4.04	106.71	177.17	2.25	1.66	187.08	2.53	3.08	0.01
3	100.17	148.53	0.6	1.48	92.6	133.3	0.64	1.44	170.38	87.97	2.67	0.52
4	96.33	138.99	0.87	1.44	194.11	67.27	2.2	0.35	177.95	86.43	2.11	0.49
5	71.09	299.8	0.6	4.22	136.24	248.98	2.06	1.83	106.4	286.74	1.8	2.69
6	n/a	n/a	n/a	n/a	231.74	109.62	2.34	0.47	246.75	61.39	2.32	0.25
7	13.34	2.99	2.33	0.22	26.96	5.59	2.39	0.21	28.49	4.66	2.4	0.16
8	83.18	412.04	0.65	4.95	182.56	344.43	1.8	1.89	130.76	244.89	1.84	1.87
9	26.21	218.3	0.16	8.33	195.93	56.86	2.56	0.29	250.1	211.64	2.71	0.85
10	36.61	299.17	0.2	8.17	138.12	216.88	1.72	1.57	166.44	210.7	1.75	1.27
11	131	351.68	0.68	2.68	244.19	267.25	2.17	1.09	250.65	259.73	2.13	1.04
12	19.77	218.37	0.15	11.05	112.84	138.95	1.87	1.23	131.08	124.24	1.8	0.95
13	168	212.53	0.76	1.27	188.16	126.07	1.95	0.67	235.22	122.6	2.22	0.52
14	131.5	202.73	0.35	1.54	178.75	134.08	2.19	0.75	227.4	201.11	2.45	0.88
15	121.61	120.66	1.08	0.99	210.45	88.82	2.08	0.42	234.9	89.3	2.25	0.38
16	43.4	388.25	0.56	8.95	115.63	303.25	1.76	2.62	124.95	499.48	1.65	4

^1^ True lumen dimension at PMC (cm). TL, true lumen; FL, false lumen; PMC, the plane of maximum compression; FLI, false lumen index.
